# Thermally induced micro-motion by inflection in optical potential

**DOI:** 10.1038/s41598-017-01848-4

**Published:** 2017-05-10

**Authors:** Martin Šiler, Petr Jákl, Oto Brzobohatý, Artem Ryabov, Radim Filip, Pavel Zemánek

**Affiliations:** 1 Institute of Scientific Instruments of CAS, Královopolská 147, 612 64 Brno, Czech Republic; 2 Charles University, Faculty of Mathematics and Physics, Department of Macromolecular Physics, V Holešovičkách 2, 180 00 Praha 8, Czech Republic; 3 Department of Optics, Palacký University, 17, listopadu 1192/12, 771 46 Olomouc, Czech Republic

## Abstract

Recent technological progress in a precise control of optically trapped objects allows much broader ventures to unexplored territory of thermal motion in non-linear potentials. In this work, we exploit an experimental set-up of holographic optical tweezers to experimentally investigate Brownian motion of a micro-particle near the inflection point of the cubic optical potential. We present two complementary views on the non-linear Brownian motion. On an ensemble of stochastic trajectories, we simultaneously determine (i) the detailed short-time position statistics and (ii) the long-distance first-passage time statistics. We evaluate specific statistical moment ratios demonstrating strongly non-linear stochastic dynamics. This is a crucial step towards a possible massive exploitation of the broad class of complex non-linear stochastic effects with objects of more complex structure and shape including living ones.

## Introduction

Biological molecular machines (or Brownian motors) utilize non-linearity and asymmetry of their free-energy landscapes to produce a useful directed motion in violently fluctuating biological environments^1, 2^. Harnessing efficiently energy from their surroundings, they are capable of producing useful work, playing a decisive role in fundamental intracellular processes^3^. Despite long-term experimental development in this field, so far, complexity of the underlying free-energy landscapes prevents a detailed understanding of their working principles^4^. To this end, it turned out to be useful to develop simple stochastic systems intended to capture individual aspects of action of such machines^1, 4, 5^. A frequently used model comprises an over-damped Brownian particle moving in an asymmetric non-linear potential. In such models, the main emphasis was put on the symmetry arguments without any particular attention paid to a specific functional form of the potential. Contrary to this, in the present work, we report for the first time an experimental observation of the conversion of the thermal noise to a directed motion dictated by a specific type of the non-linearity with an inflection point. We believe that such conversion of stochastic behaviour to a local motion can be advantageously exploited to develop new thermal ratchets and advanced thermal motors. The reported experiments were performed using holographic optical tweezers^6–12^, showing thus that this widely accessible platform can be used successfully for experimental exploration of the whole spectrum of optical potential landscapes^13^ including non-linear ones^14, 15^. We believe that it will open many future possibilities to experimentally develop new highly non-linear Brownian motors efficiently harnessing energy from their fluctuating environments^5, 16, 17^ as well as in biological matter^18^.

Analysis of non-linear stochastic dynamics requires simple and reliable techniques for a rather limited number of Brownian trajectories. The non-linear Brownian motion can be analytically characterized (i) by a short-time statistics of the particle position which offers a detailed view on unexplored local dynamics. In a complementary way, (ii) by a global, large-distance first-passage time statistics^19^, which is crucial for understanding e.g. rates of chemical processes^20^, phase transitions and passages through bifurcations^21, 22^, and macroscopic transport properties of complex systems^23–25^. Both these approaches provide complementary pictures of the non-linear Brownian motion and in particular, they allow to understand beneficial global and local effects of thermal noise on the stochastic dynamics^26^. To gain relevant statistical insight, at least comparisons of mean values and variances of the two quantities should be analysed for experimentally available number of trajectories. It can be highly expected that such experimental analysis and its comparison with theory can start really wide-range investigation of non-linear thermal Brownian motion and its application in thermal engines.

## Results

**Figure 1:**
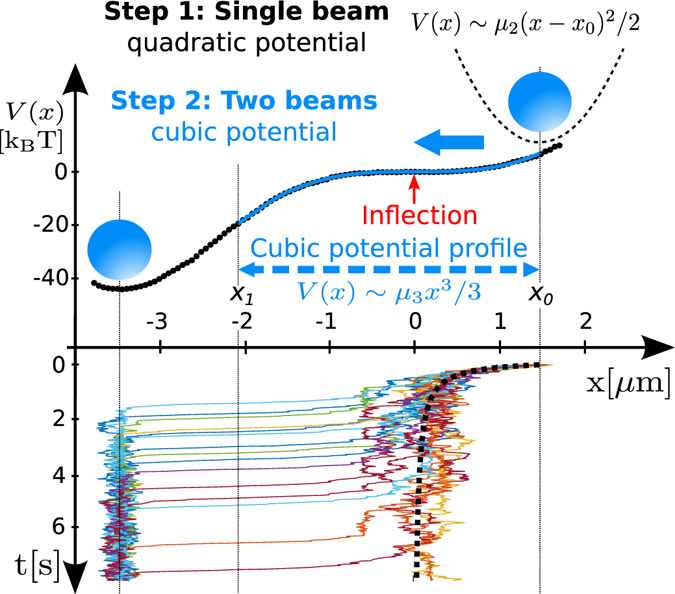
Principle of the experiment and examples of measured trajectories. Step 1: The particle (blue spot) is trapped in a single beam and follows Brownian motion in a quadratic potential close to its bottom. Step 2: The single beam is replaced with two co-propagating beams displaced in *x*-axis to form a non-linear single-well potential (dotted) with a cubic part (blue) along *x*-axis. Consequently the particle moves down the potential well (indicated by a blue arrow) passing through the cubic part and reaching the global minimum. Then the two beams are replaced with a single beam which confines the particle near the former global minimum and transports it to the initial position *x*_0_. Steps 1 and 2 are repeated with the same particle to get a statistical ensemble. The dotted curve denotes the reconstructed potential profile (in *k*_*B*_ *T* units) from the particle trajectories and a fit to the cubic potential profile gives *μ*_3_ = (6.65 ± 0.05)*k*_*B*_ *T*/*μ*m^3^. A few measured trajectories are plotted as colour curves at the bottom part of the figure and the dashed one shows the deterministic trajectory without the influence of Brownian motion corresponding to *T* = 0.

Any non-linear potential can be locally approximated using Taylor series and therefore basic local potentials are described by $V(x)\sim {\mu }_{n}{x}^{n}/n$, where *n* is a natural number. Complex noise-induced effects arise from a linear combination of these basic potentials. In order to understand the effects and potentially exploit them to design molecular motors, it is necessary to focus attention on individual terms of the series, which is challenging to isolate in current experiments^15^. The first in a line of non-linear potentials, the strong cubic potential $V(x)\sim {\mu }_{3}{x}^{3}/3$, already yields a stochastic dynamics with a number of intriguing properties. It induces the over-damped non-linear motion, for which both the position *x* and the first-passage time *τ* depends on the temperature *T* of the environment in a universal, but different way. This asymmetric potential is unbounded with an unstable inflection point at the origin *x* = 0, as is visible from experimentally achieved cubic optical potential in Fig. 1. Close to the inflection point, the potential energy is constant and forms a plateau where the particle starting at *x* > 0 slows down in the over-damped regime. Without thermal noise (*T* = 0, deterministic motion), the over-damped particle is unable to pass through the inflection point at *x* = 0 and remains there as the dashed deterministic trajectory in Fig. 1 illustrates. Stochastic dynamics (*T* > 0) is apparently distinct from the deterministic one as is apparent from measured trajectories shown in Fig. 1. In this case, the thermal noise prevails over the potential energy and the particle diffuses freely across the vicinity of the inflection point and then it gradually accelerates for *x* < 0. Away from this region, the potential is much stronger as compared to *k*_*B*_ *T* and the particle motion becomes fast and nearly deterministic even when *T* > 0 (sections of trajectories for *x* < −1 *μ*m in Fig. 1). Therefore, only the region near the inflection point is relevant for the thermally-induced directed particle motion.

Here, we report on experimental investigation of the both complementary aspects of particle motion in the cubic optical potential using routine and broadly available setup of holographic optical tweezers suitable for different objects. The proposed method uses dynamic modification of the intensity profile of the trapping beam from quadratic (i.e., the classical single beam optical trap) to cubic formed by two properly designed co-propagating Gaussian beams^27^.

### Short-time evolution of stochastic motion

The non-linear stochastic motion of the particle near the inflection point at *x* = 0 can be described by the statistics of *x*(*t*) for small time *t*, until the particle feels a strong force from the cubic potential. For the short time, a formal integration of the Langevin equation

1 $$\frac{{\rm{d}}x}{{\rm{d}}t}=-\,\kappa {x}^{2}(t)+\sqrt{\frac{2{k}_{B}T}{\gamma }}\xi (t),$$

gives a local insight. Above, *ξ*(*t*) is the standard Gaussian white noise (〈*ξ*(*t*)〉 = 0, 〈*ξ*(*t*)*ξ*(*t*′)〉 = *δ*(*t* − *t*′)), *κ* = *μ*_3_/*γ*, and *γ*, *k*_*B*_, *T* denote the hydrodynamic drag coefficient, the Boltzmann constant and the absolute temperature, respectively. After the formal integration of equation (1) in an interval of time (*t*_0_, *t*), we obtain

2 $$x(t)=x({t}_{0})-\kappa {\int }_{{t}_{0}}^{t}{x}^{2}({t}_{1}){\rm{d}}{t}_{1}+\sqrt{\frac{2{k}_{B}T}{\gamma }}{\int }_{{t}_{0}}^{t}\xi ({t}_{1}){\rm{d}}{t}_{1}.$$

Iterating this formal solution up to the second order, we get (assuming further *t*_0_ = 0)

3 $$x(t)=x(0)-\kappa {\int }_{0}^{t}{[x(0)-\kappa {x}^{2}(0){t}_{1}+\sqrt{\frac{2{k}_{B}T}{\gamma }}{\int }_{0}^{{t}_{1}}\xi ({t}_{2}){\rm{d}}{t}_{2}]}^{2}{\rm{d}}{t}_{1}+\sqrt{\frac{2{k}_{B}T}{\gamma }}{\int }_{0}^{t}\xi ({t}_{1}){\rm{d}}{t}_{1},$$

which yields the following short-time approximation for the mean position

4 $$\langle x(t)\rangle \simeq \langle x\mathrm{(0)}\rangle -\kappa \langle {x}^{2}\mathrm{(0)}\rangle t-\kappa \frac{{k}_{B}T}{\gamma }{t}^{2}+{\kappa }^{2}\langle {x}^{3}\mathrm{(0)}\rangle {t}^{2}.$$

**Figure 2:**
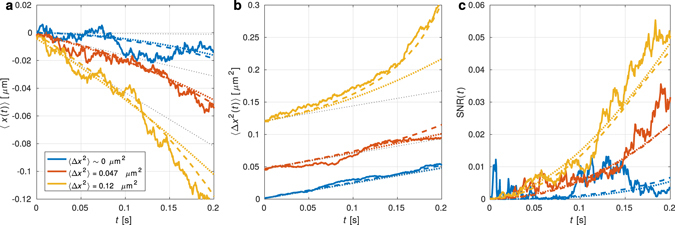
Experimental evidence of short-time stochastic non-linear dynamics. Panel (**a**) shows the mean particle position 〈*x*(*t*)〉, panel (**b**) its variance 〈Δ*x* ^2^(*t*)〉 and panel (**c**) the signal-to-noise ratio SNR(*t*) = 〈*x*(*t*)〉^2^/〈Δ*x* ^2^(*t*)〉 developing in time from 〈*x*(0)〉 = 0 for three initial variances 〈Δ*x* ^2^(0)〉 = 0 (blue), 0.047 (red), and 0.12 *μ*m^2^ (yellow). Solid curves correspond to experimental data, dotted to equations (4, 5) in (**a**) and (**b**) or their ratio in **(c)** for *κ* obtained from the potential profile and corresponding hydrodynamic drag coefficient. Dotted grey lines show contribution of only linear time dependence terms taking place in equations (4) and (5). Dashed curves correspond to Monte Carlo simulations of the particle motion based on numerical solution of equation (1) with initial Gaussian distribution.

The cubic non-linearity *κ* causes on average a unidirectional non-linear drift of the particle evolving linearly in time *t*. When 〈*x*(0)〉 = 0, the particle mean position depends dominantly on the initial variance 〈*x* ^2^(0)〉 and temperature *T* of the environment. Figure 2a fully supports these theoretical conclusions with experimental observations and stochastic simulations using equation (1). Further, for an initial position very close to the origin ($x\mathrm{(0)}\simeq 0$, blue curves in Fig. 2a), the mean particle position spontaneously starts to change much slower (as ∼ *t* ^2^) for a short time and it is purely triggered by the environmental thermal noise.

The short-time effect of the thermal noise on the variance of the particle position, 〈Δ*x* ^2^(*t*)〉 = 〈[*x*(*t*) − 〈*x*(*t*)〉]^2^〉, is rather similar to the case of a free Brownian motion^28^,

5 $$\langle {\rm{\Delta }}{x}^{2}(t)\rangle \simeq \langle {\rm{\Delta }}{x}^{2}\mathrm{(0)}\rangle +2\frac{{k}_{B}T}{\gamma }t+8{\kappa }^{2}{t}^{2}{\langle {\rm{\Delta }}{x}^{2}\mathrm{(0)}\rangle }^{2}.$$

Non-linearity of the potential starts to manifest itself at longer time (the term ∼ *t* ^2^) and only through a non-trivial dependence on the initial variance. The blue and red curves in Fig. 2b show almost perfect linear time dependence as in the case of classical free diffusion while the yellow curve exhibits non-linear behaviour driven by the initial variance.

One of the important measures of the presence of noise-to-signal transition is the raising value of the signal-to-noise ratio SNR(*t*) = 〈*x*(*t*)〉^2^/〈Δ*x* ^2^(*t*)〉^28^. It compares the mean displacement, which quantifies the average directed motion, to a possible deviation from this mean value, described by the variance. Figure 2c reveals that the SNR obtained for larger initial variances (red and yellow curve) increases in time much faster (as ∼ *t* ^2^) comparing to the case of vanishing initial variance (blue curve) following ∼*t* ^3^. This corresponds to the signal-to-noise ratio continuously powered by the environmental Brownian noise (∼*t* ^3^) or only by the initial noise (∼*t* ^2^)^28^. In all cases demonstrated in Fig. 2, the coincidence of experimental and theoretical curves is very persuading. Further, one may see that the linear approximation is sufficient up to time 50 ms (best coincidence of the linear term is for the yellow curve with the largest initial variance) while for later times the quadratic and higher terms are necessary. This is further shown by difference between simulated (dashed) and theoretical (coloured dotted) curves where the significant difference appears for *t* > 0.1 s. For *t* > 0.1 s even the quadratic term from equation (5) is insufficient for large initial variance (yellow curve) and the experimental data follow simulated ones only. Notice that, due to the cubic potential, both absolute values of the means 〈*x*(*t*)〉 and SNR(*t*) increase more rapidly in time for the larger initial variance 〈Δ*x* ^2^(0)〉.

**Figure 3:**
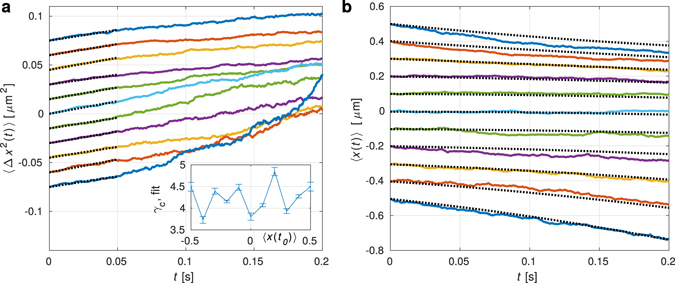
Short time evolution of variance 〈Δ*x* ^2^(*t*)〉 and the mean particle position 〈*x*(*t*)〉. The initial positions of particle trajectories 〈*x*(0)〉 were equally placed at 0.5, 0.4, …, −0.5 *μ*m (from bottom to top). (**a**) Short time evolution of variance 〈Δ*x* ^2^(*t*)〉. Thick curves show experimental data (vertically displaced with respect to each other, offset 0.15 × 〈*x*(0)〉) and thin dotted curves correspond to equation (5) fitted to experimental data with the only fitting parameter *γ*_*c*_ (see Methods). Inset shows *γ*_*c*_ obtained this way for initial particle position. (**b**) Short time evolution of the mean particle displacement from the starting location 〈*x*(0)〉. Thick curves show experimental data and thin lines are obtained using equation (4) with already obtained values of *γ* and *κ*.

Figure 3a shows the experimental records of variance 〈Δ*x* ^2^(*t*)〉 evolution in time for cases where the initial positions *x*(0) of each trajectory correspond to 0.5, 0.4, …, −0.5 *μ*m. Due to this assumption the initial variance of the particle positions 〈Δ*x* ^2^(0)〉  ﻿is zero﻿, however, in Fig. 3a the curves are mutually shifted (by offset 0.015 *μ*m^2^) to better distinguish individual trajectories. Figure 3a clearly demonstrates that variance indeed increases linearly for *t* < 50 ms. In order to describe a longer process one would need at least 3^rd^ order Taylor expansion of variance 〈Δ*x* ^2^(*t*)〉 since the initial variance is zero and the quadratic term in equation (5) vanish. The particle trajectories starting at different locations are plotted in Fig. 3b and they coincide with the theoretical predictions over almost four times longer period comparing to variances in Fig. 3a. The colours and positions of the curves correspond to each other in both Fig. 3a and b and the light-blue curves for initial position *x*(0) = 0 correspond to blue curves in Fig. 2.

### First-passage time analysis

The global insight into the strongly non-linear dynamics is provided by the long-distance passage time statistics. The *first-passage time* (FPT) *τ*(*x*_0_ → *x*_1_) is the random time when the Brownian particle starting at *x*_0_ and moving in the potential *V*(*x*) reaches the position *x*_1_ for the first time. The mean first-passage time^29^ is given by

6 $$\langle \tau ({x}_{0}\to {x}_{1})\rangle =\frac{\gamma }{{k}_{B}T}{\int }_{{x}_{1}}^{{x}_{0}}d{y}_{1}{\int }_{{y}_{1}}^{\infty }d{y}_{2}\,{{\rm{e}}}^{-[V({y}_{2})-V({y}_{1})]/{k}_{B}T}.$$

**Figure 4:**
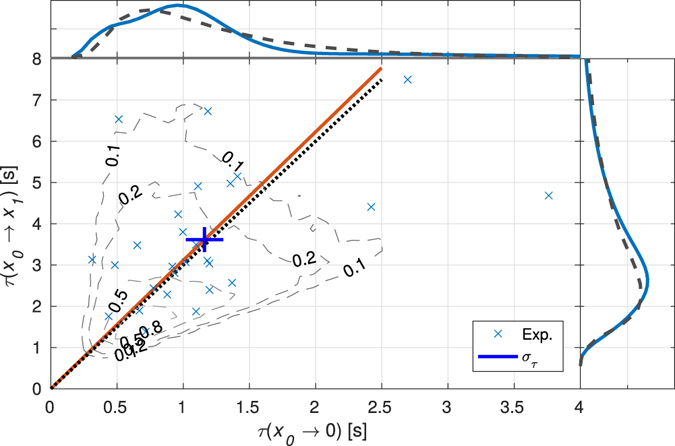
First-passage times and the ratio *R* = 〈*τ*(*x*_0_ → 0)〉)/(〈*τ*(*x*_0_ → *x*_1_)〉. Light blue × marks the first-passage time experimentally determined for each trajectory. The intersection of thick blue lines depicts the mean first-passage times 〈*τ*(*x*_0_ → 0)〉 (*x*-axis) and 〈*τ*(*x*_0_ → *x*_1_)〉 (*y*-axis) and the lengths of blue lines denote standard errors of the both means, i.e. ${[\frac{1}{N(N-1)}\sum {({\tau }_{i}-\langle \tau \rangle)}^{2}]}^{1/2}$. Full red line passing through the blue cross and the dotted black line depict the measured ratio *R* = 0.32 and theoretically predicted value of *R* = 1/3, respectively. Comparison of experimental data and the computer simulation is shown by background contours revealing 2D histogram constructed using the both first-passage times (maximum of histogram normalized to 1). Considered trajectories start at *x*_0_ = 1.2 *μ*m and end in 0 or *x*_1_ = −2.1 *μ*m, see Fig. 1. Side panels show the first-passage time probability density function (blue curves) applying the kernel smoothing function on the experimental data and the probability density function calculated from the simulated data (dashed curves).

We have measured the FPT *τ*(*x*_0_ → *x*_1_) needed to pass through the cubic non-linearity centred at its inflection at *x* = 0 for a particle that departs from *x*_0_ > 0, and passes to *x*_1_ < 0. Further, we have also determined the second FPT, *τ*(*x*_0_ → 0), accordingly. Interestingly enough, the relative mean first-passage times approach universal values dependent only on the order of the non-linear potential, giving for the cubic one^30^:

7 $$R=\frac{\langle \tau ({x}_{0}\to \mathrm{0)}\rangle }{\langle \tau ({x}_{0}\to {x}_{1})\rangle }\to \frac{1}{3},\,1-R=\frac{\langle \tau \mathrm{(0}\to {x}_{1})\rangle }{\langle \tau ({x}_{0}\to {x}_{1})\rangle }\to \frac{2}{3},$$

when both *x*_0_ and *x*_1_ are far away from the inflection point. They reflect the basic feature of the cubic potential, that the directed particle motion is slowed down near the inflection point. This fact is clearly visible on the sample of measured trajectories shown in Fig. 1, where there are several long-living trajectories spending considerable amount of time crossing the inflection point back and forth. Figure 4 demonstrates a very good agreement between the theoretical prediction *R* = 1/3 and the experimental value *R* = (0.32 ± 0.05) even though the starting and final points are far from infinity and asymmetrically placed with respect to the inflection point.

Since the FPT is a stochastic quantity, its mean should be directly compared to its variance by a signal-to-noise ratio^30^ (SNR_*τ*_)

8 $${{\rm{S}}{\rm{N}}{\rm{R}}}_{\tau }=\frac{\langle\tau ({x}_{0}\to {x}_{1})\rangle}{{\langle{\rm{\Delta }}{\tau }^{2}({x}_{0}\to {x}_{1})\rangle}^{1/2}}{\rm{w}}{\rm{i}}{\rm{t}}{\rm{h}}\mathop{{\rm{l}}{\rm{i}}{\rm{m}}}\limits_{\mathop{{x}_{1}\to -{\rm{\infty }}}\limits^{{x}_{0}\to {\rm{\infty }}}}{{\rm{S}}{\rm{N}}{\rm{R}}}_{\tau }=\sqrt{3},$$

which quantifies the relative significance of the two characteristics. For the experimental data presented above we determined slightly different value SNR_*τ*_ = 2.3 ± 1.6. The large error is caused by large variations of measured first-passage times. In order to confirm theoretically predicted behaviour we simulated random particle motion in 10^4^ independent trajectories. The simulated SNR_*τ*_ corresponded perfectly to $\sqrt{3}$ ≈ 1.73 for almost infinite interval of particle motion and we obtained value of 1.62 when considering experimental positions in the range 〈*x*_0_, *x*_1_〉. Further, final duration of the measurement *t*_max_ ≪ ∞ leads to stronger deviation of the SNR_*τ*_ from its ideal theoretical value because certain experimental trajectories did not develop behind *x*_1_. We found out that both the mean first-passage time and the square root of variance scale differently with *t*_max_. As a result, SNR_*τ*_ exceeds $\sqrt{3}$ for *t*_max_ ≲ 15 s. For the experimentally used *t*_max_ = 8 s simulations predicted SNR_*τ*_ = 2.32 which is almost in the centre of the acquired experimental interval SNR_*τ*_ = 2.3 ± 1.6.

The presented statistics of first-passage times allows meaningful description of a stochastic motion for longer times in the unstable potential in contrast to the time-dependent moments of position described in previous section because it stops rapidly diverging trajectories at *x*_1_ which in fact serves as the absorbing boundary.

## Conclusions

We have demonstrated two complementary views on a Brownian motion in the cubic potential which models e.g. firing neurons^22^ and molecular motors^23^. The potential shows unique universal global characteristics, as well as revealing local properties describing non-linear transformation of thermal noise into a directed motion over an unstable inflection point. We have shown that all these principal properties of barrier-less transitions are robust enough to be observed in a widely accessible experimental platform, making them a good candidate for further thorough exploration and utilization in non-linear thermal motors, testing basic principles of stochastic dynamics and thermodynamics^31^. Very prospective and challenging is a venture to the under-damped regime, which now becomes experimentally accessible^15, 32^ and later, to the quantum regime, which is currently under active investigation^33–35^.

## Methods

### Experimental setup

The optical potential landscape containing a cubic part was formed using holographic optical tweezers^36–38^. The beam intensity is shaped into the appropriate lateral profile using the spatial light modulator (SLM) having 512 × 512 liquid crystal pixels. If the phase mask is changed on the SLM, liquid crystals need some time (about 18 ms) to reorient to new direction. During this period the phase mask, and thus the laser beam intensity profile, is not well defined. Therefore, using the acousto-optical deflector (AOD) we blocked the beam at SF1 during this period so that it did not interact whit the particle. The laser beam is enlarged 6× by the telescope L1-L2 to overfill the SLM chip. Both the AOD and SLM are operated in the first diffraction order while the zero orders are blocked by spatial filters SF1 and SF2, respectively. The laser beam is focused into the sample chamber with microscope objective (magnification 40x, numerical aperture 0.65) giving a beam waist radius 2 *μ*m.

The sample chamber consists of two coverslips separated by foil spacers 90 *μ*m thick and the sample itself is composed of polymer spherical particles of (994 ± 10) nm in diameter (Duke Standards 41303, Thermo Scientific) dispersed in distilled water with 1% of surfactant sodium-dodecyl-sulphate (SDS) to prevent sticking of microobjects to the coverslip. The sample is attached to pizeo-stage which is used to overlap the selected particle with the optical trap. Once the particle is trapped laterally in the beam it is pushed axially against the coverslip surface. The sample is illuminated by a Köhler illumination system and observed with the fast Basler camera providing acquisition rate of 2000 fps on the limited field of view 256 × 40 pixels.

### Measurement protocol

**Figure 5:**
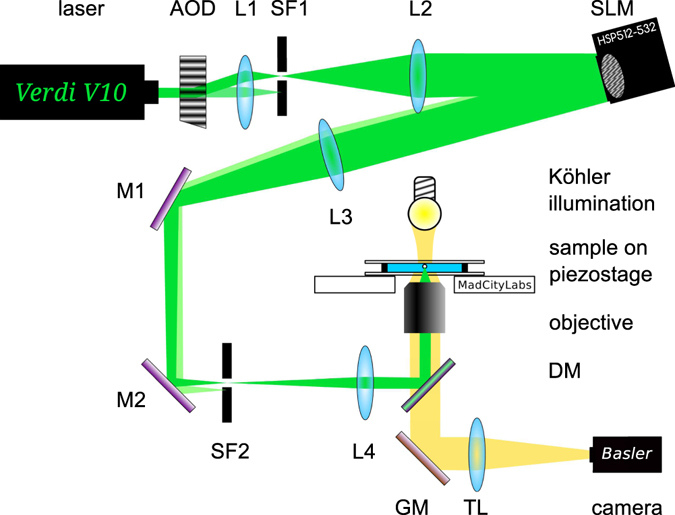
Experimental setup. Laser – Verdi V10 (Coherent, vacuum wavelength 532 nm), AOD – acousto-optical deflector R35085-3 (Gooch&Housego), lens L1 – AC254-050-A (f_1_ = 50 mm, Thorlabs), spatial filter SF1 – high-power precision pinhole P25C (*ϕ*25 *μ*m, Thorlabs), lens L2 – AC254-300-A (f_2_ = 300 mm, Thorlabs), SLM – spatial light modulator HSP512-532-PCIe (Meadowlark Optics), lens L3 – AC508-500-A (f_3_ = 500 mm, Thorlabs), M1, M2 – broadband dielectric mirrors BB1-E02 (Thorlabs), spatial filter SF2 – iris aperture SM1D12D (Thorlabs), lens L4 – AC254-150-A (Thorlabs), DM - dichroic mirror (made in-house), objective – PlanC N 40x/0.65 (Olympus), combined motorized microstage and piezo-driven nanostage – NanoView (MadCityLabs), GM – protected gold mirror PF10-03-M01 (Thorlabs), tube lens TL – AC254-200-A (Thorlabs) and camera acA640-750-um (Basler). The Köhler illumination is built with IR filter to prevent sample heating.

The cubic profile of the optical potential landscape was achieved by combination of two spatially separated Gaussian beams with slightly different intensities (see Fig. 1). The lateral distance between the two Gaussian focal points was 3.9 *μ*m, beam waists of both focused beams were 2 *μ*m and the intensity of one beam was one half of the other. In order to localize more precisely the particle in the direction perpendicular to the non-linear laser profile, the lateral intensity profiles were squeezed here providing elliptical lateral beam profiles. Their major axes were oriented along the axis where the non-linear potential was formed (see an example of the phase mask on the SLM in Fig. 5).

The measurement procedure starts with the step 1 where the single beam optical trap (SBT) is formed at the starting position of the trajectory (see Fig. 1) and the particle is confined in this quadratic potential well. Before the step 2 the laser beam is blocked with AOD and the phase grating generating cubic potential landscape is placed on the SLM. After 18 ms, corresponding to realignment of the liquid crystals, the beam is unblocked and the particle trajectory in the cubic potential is recorded for 8 s with the CCD camera frame rate 2 kHz. The laser beams forming the cubic potential are switched off and replaced with the single beam focused to the former potential minimum. The particle is trapped and delivered in the single beam to the starting location and the whole procedure is repeated.

### Data processing

The particle trajectories were obtained from the recorded images using 2D correlation between the recorded frames and a particle image. The final particle positions were determined as the centre of the Gaussian fit (see trajectories in Fig. 1).

### Influence of the surface

As the particle moves in close vicinity of a surface, the no-slip boundary condition influences the streamlines of fluid motion. This effectively increases the drag coefficient and, further, causes anisotropy of motion in directions parallel and perpendicular to the surface^39^. Therefore, the drag coefficient in the direction parallel to the surface can be described as *γ* = *γ*_0_ *γ*_*c*_(*h*/*r*) where *γ*_0_ = 6*πηr* is the drag coefficient far from the surface and *γ*_*c*_ is the correction factor dependent on the ratio of particle radius *r* and separation *h* between the particle centre and the surface. If a particle is trapped in optical tweezers (i.e. in all three-directions) in the surface vicinity, the measured values of *γ*_*c*_ are between 2–3^40^. Since in our geometry we use loosely focused laser beams that push the particle against surface, we can expect values of *γ*_*c*_ even bigger than 3. It is important to note that the coefficient *κ* (or *μ*_3_) was obtained by analysis of motion close to the surface and its value already includes the influence of surface. Therefore, the unknown coefficient *γ*_*c*_ explicitly takes part only in the random motion term in equation (1) and in quadratic or linear terms of equations (4) or (5), respectively. We fitted the equation (5) to the data presented in Fig. 3a and determined the fitting parameter *γ*_*c*_ for each initial position (see the inset in Fig. 3a). The mean value for all shown initial positions is equal to *γ*_*c*_ = 4.2 ± 0.3.

Furthermore, *γ*_*c*_ can be complementary obtained using the distribution of first-passage times, see Fig. 4. In order to find it, we analysed 5 × 10^4^ particle trajectories obtained from equation (1) by Monte Carlo computer simulations using the same value of *κ* but with different *γ*_*c*_. From collected trajectories corresponding to the same *γ*_*c*_, we determined first-passage times and constructed the probability density function (PDF) describing times *τ*(*x*_0_ → *x*_1_). Using the kernel smoothing function we constructed the same PDF for experimental data, see dashed curve in the right panel of Fig. 4. Further, we searched for the closest match between simulated and experimental PDFs using least-squares fitting and we obtained the following value *γ*_*c*_ = (4.6 ± 0.2). Values of *γ*_*c*_, obtained by both methods, confirm a very good coincidence.

### Determination of the particle mean position and variance for different starting conditions

The results presented in Figs 2 and 3 took advantage of multiple particle passages through the same *x* position in following protocol:

1. For a given trajectory we find the time *t*_0_ when the particle reaches the selected point *x*_0_ for the first time.
2. We select data points of the analysed trajectory in the following time period of length 0.2 s lasting from *t*_0_ to *t*_0_ + 0.2.
3. We search for another crossing of the particle trajectory with *x*_0_ (point 1) at *t* > *t*_0_ + 0.2.
4. If no such crossing is found, another trajectory is taken for the same analyses as in 1 and 2 above.
5. We calculate the mean particle position and variance from data points collected in 1–3 above.
6. The procedure 1–5 is repeated for different initial positions *x*_0_.
